# High-Fat Diet-Induced Mild Obesity Alters the Activation of T Cells and Maintains Intestinal Homeostasis in Food Allergy Animal Model

**DOI:** 10.3390/foods14111852

**Published:** 2025-05-23

**Authors:** Fan Yang, Ruofan Xie, Meijia Huang, Chunqiu Hu, Yong Wu, Xin Li, Hongbing Chen

**Affiliations:** 1 College of Food Science & Technology, Nanchang University, Nanchang 330047, China; 18720995289@163.com (F.Y.); ncuspyxieruofan@163.com (R.X.); ericyo918@hotmail.com (Y.W.); 2 State Key Laboratory of Food Science and Resources, College of Food Science & Technology, Nanchang University, Nanchang 330047, China; 3 Jiangxi Province Key Laboratory of Food Allergy, Nanchang University, Nanchang 330047, China; 4 School of Food Science, Henan Institute of Science and Technology, Xinxiang 453003, China; huangmj@hist.edu.cn; 5 Department of Toxicology, School of Public Health, Anhui Medical University, Hefei 230032, China; huchqiu@163.com; 6 Sino-German Joint Research Institute, College of Food Science & Technology, Nanchang University, Nanchang 330047, China

**Keywords:** food allergy, high-fat diet, immune response, intestinal barrier

## Abstract

A close association between obesity and food allergies has been described, but from the perspective of T cell differentiation, controversial findings exist regarding the impact of high-fat diet (HFD) feeding on the development of food allergies. Our study aimed to explore the relationship between HFD-induced mild obesity and food allergy development in female BALB/c mice. Following 18 weeks of HFD feeding, experimental animals demonstrated a 10.92% body weight gain accompanied by a 32.45% elevation in total cholesterol levels and a 39.50% increase in low-density lipoprotein–cholesterol levels. Upon allergen challenge, control diet (COD)-fed mice developed obvious food allergy symptoms and hypothermia, which were slightly alleviated in HFD-fed mice. Flow cytometry revealed that splenocytes from HFD-fed mice exhibited a 102.32% increase in CD4^+^T-bet^+^ T (Th1) cell populations and a 38.69% elevation in CD4^+^RORγt^+^ T (Th17) cell populations compared to COD-fed allergic mice. These changes corresponded with a 28.41% reduction in the Th2/Th1 ratio and a 56.67% increase in the Th17/Treg ratio. Moreover, real-time quantitative PCR showed that HFD-fed allergic mice had higher expressions of *Foxp3*, *Occludin1*, and *TJP1* in the intestine. Therefore, we concluded that HFD-induced mild obesity restored the Th2/Th1 and Th17/Treg balance, reinforced intestinal barrier function, and thereby did not increase allergy risk in female BALB/c mice.

## 1. Introduction

Food allergies have skyrocketed in prevalence in recent decades, currently affecting an estimated 7.6% of children and 10.8% of adults worldwide [1]. A large epidemiological study (1998–2018) revealed that hospitalization rates for food allergies among young children in the United Kingdom increased by 6.6% annually [2]. Recent Chinese research demonstrated a surge in childhood food allergy prevalence in Chongqing, rising from 3.5% to 11.1% between 1999 and 2019 [3]. Growing evidence links allergy risk to environmental and dietary shifts, particularly the dramatic transition in dietary macronutrient composition observed over recent decades, from high-carbohydrate, high-fiber diets to predominantly high-fat diets (HFDs). The “Dietary Fat Hypothesis”, first proposed by Gideon Lack, postulates that fatty acids in modern diets may enhance allergic susceptibility [4,5].

The excessive consumption of an HFD promotes obesity due to high caloric density. Epidemiological studies consistently identify overweight and obesity as significant risk factors for allergic pathogenesis [6,7]. Hussain et al. demonstrated that HFD-induced severe obesity exacerbates food allergy-associated atopic dermatitis, mediated by fatty acid-driven gut microbiota dysbiosis [8]. HFD-derived free fatty acids disrupt intestinal barrier integrity and cause intestinal inflammation during gastrointestinal digestion, which in turn potentiates allergic sensitization [9]. Moreover, an HFD is characterized by elevated saturated fatty acids (SFAs) and n-6 polyunsaturated fatty acids (PUFAs) coupled with reduced n-3 PUFA levels, a lipid profile that predisposes a person to allergic sensitization [10,11]. Specifically, n-6 PUFAs drive prostaglandin E2 synthesis, suppressing IFN-γ production while enhancing IL-4 production in T cells [12,13]. Nonetheless, there is a lack of rigorous research data to demonstrate the effect of an HFD and its induced mild obesity (about 20% weight gain) on food allergies.

Food allergy is associated with dysregulated T cell differentiation, where patients exhibit Th2-skewed immune responses that disrupt Th1/Th2 homeostasis [14]. Previous research indicates that obesity promotes Th1 polarization, which modulates Th2 responses [15]. Moreover, emerging evidence highlights Th17/Treg balance in regulating allergic immune response [16,17]. Elevated serum IL-17A derived from Th17 cells exacerbates inflammatory processes in non-IgE-mediated cow’s milk allergy [18]. HFD-induced obesity specifically triggers splenic Th17 activation in murine models [19]. This aligns with findings demonstrating that IL-17A-producing Th17 cell accumulation in obese mice contributes to inflammatory disorders [20], though paradoxically, Th17 activity may also potentiate cow’s milk allergy pathophysiology [21]. However, the immunomodulatory effects of HFD-induced obesity on food allergy pathogenesis through Th1/Th17 cell activation remain uncharacterized. Therefore, our study aims to elucidate the impact of HFD feeding on β-lactoglobulin (BLG)-induced allergy.

## 2. Materials and Methods

### 2.1. Mice and Feeding

Female BALB/c mice (5-week-old) were obtained from GemPharmatech Co., Ltd. (Certificate No. SCXK 2021–0004), and housed under specific pathogen-free (SPF) conditions with controlled temperature (22 ± 1 °C), humidity (55 ± 5%), and 12 h light/dark cycles. Following a 7-day acclimatization period, experimental protocols commencing at 6 weeks of age involved ad libitum access to the control diet (COD; 10% from fat, Jiangsu Xietong Pharmaceutical Bio-engineering Co., Ltd., Nanjing, China) or high-fat diet (HFD; 60% from fat, Jiangsu Xietong Pharmaceutical Bio-engineering Co., Ltd., Nanjing, China) for 18 consecutive weeks. The compositions of the HFD and COD are shown in Table S1. All mice were kept in compliance with the guidelines set by the Center of Laboratory Animal Science at Nanchang University, and the experiments were conducted under an ethical animal research license (NCULAE-20240229002).

### 2.2. Experimental BLG-Induced Food Allergy Animal Model

**Figure 1:**
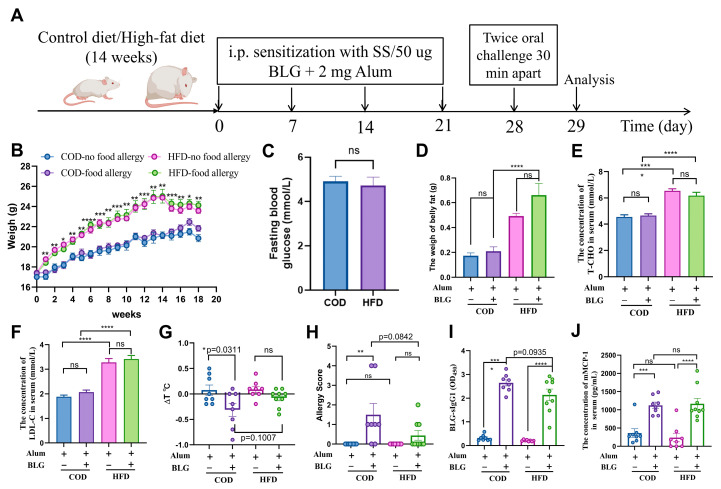
The change in allergic immune response in mice fed with an HFD. (**A**) Experimental design of obesity and food allergy induced by BLG in mice. (**B**) Body weight gain. (**C**) The fasting blood glucose. (**D**) The weight of belly fat. (**E**) The total cholesterol levels in the serum. (**F**) The low-density lipoprotein levels in the serum. (**G**) Changes in body temperature (∆T) of mice with or without food allergy fed a COD or HFD. (**H**) Allergy symptom score. (**I**) Serum BLG-specific IgG1 levels. (**J**) Serum mMCP-1 levels. i.p. = intraperitoneal injection; HFD = high-fat diet; COD = control diet; BLG = β-lactoglobulin. Error bars indicate means ± SEMs; SS = sterile saline; ns = no significant. * *p* < 0.05, ** *p* < 0.01, *** *p* < 0.005, and **** *p* < 0.001.

The BLG-induced food allergy model was established with modifications from a previous protocol [22]. Briefly, as shown in Figure 1A, mice in the food allergy group fed with the COD (*n* = 8) and HFD (*n* = 9) received intraperitoneal sensitization with 200 µL of sterile saline containing 50 μg of BLG and 2 mg of Alum Adjuvant AlK(SO4)_2_·12H_2_O (Sigma-Aldrich, St. Louis, MO, USA) on the 1st, 7th, 14th, and 21st day. Mice in the non-food allergy group fed with the COD (*n* = 8) and HFD (*n* = 8) were administered 200 µL of sterile saline containing 2 mg of AlK(SO4)_2_·12H_2_O at the same time point. Twenty-eight days later, mice were treated with 200 µL of sterile saline containing 12 mg of BLG through oral gavage twice, with a 30 interval each time. Allergic symptoms were evaluated and scored for allergic symptoms post challenge according to Table S2. Meanwhile, changes in temperature were also monitored.

### 2.3. Serum Lipids Detection

To evaluate the change in serum lipid in mice, total cholesterol (T-CHO) and low-density lipoprotein–cholesterol (LDL-C) in serum were analyzed using commercial kits (Nanjing Jiancheng, Nanjing, China), following blood collection from anesthetized mice and centrifugation (1000× *g*, 20 min).

### 2.4. Serum Biomarker Quantification

Serum samples collected 18 h post challenge were analyzed by ELISA for BLG-specific immunoglobulin (Ig)G1, as described previously [23]. Briefly, 96-well ELISA plates were coated with 2.5 μg of BLG and incubated at 4 °C for 12 h. After three washes with PBS containing 0.05% Tween-20 (PBST), the plates were blocked with 250 µL of 3% gelatin (Sigma Aldrich, St. Louis, MO, USA) at 37 °C for 1 h. The serum was diluted to 1:100,000, and 100 µL of samples was added to the plate. Following a 1 h incubation at 37 °C and subsequent washes, 100 µL of 3,3′,5,5′-tetramethylbenzidine (NeoBioscience, Shenzhen, China) was adding and incubated at 37 °C for 15 min. The reaction was terminated with 50 μL of 2 M H_2_SO_4_, and absorbance was measured at 450 nm. In addition, the serum mouse mast cell peotease-1 (mMCP-1) level was determined using a commercial ELISA kit (Thermo Fisher Scientific, Shanghai, China). ELISA was conducted following the manufacturer’s protocol.

### 2.5. Cell Stimulation and Cytokines Detection

Spleen cells and mesenteric lymph nodes (MLN) cells were mechanically dissociated through gentle grinding in RPMI 1640 medium (Solarbio, Beijing, China) using sterile syringe plungers, followed by filtration through 70 μm cell strainers. Red blood cells were removed using Red Blood Cell Lysis Solution (Solarbio, Beijing, China) for 5 min at room temperature. Both MLN and spleen cells were washed twice with RPMI 1640 medium and resuspended in medium (RPMI 1640 medium supplemented with 10% FBS (Cellmax, Beijing, China) and 1% penicillin/streptomycin (Solarbio, Beijing, China)).

Those cells were seeded at 5 × 10^6^ cells/mL in cell culture plates (NEST, Wuxi, China), and stimulated with 40 μg of BLG in a humidified 5% CO_2_ incubator at 37 °C for 96 h. Supernatants were collected via centrifugation at 500× *g* for 5 min at 4 °C to remove cellular debris, then stored at −80 °C until cytokine quantification. IL-5, IL-13, IFN-γ, and IL-17A levels were determined using commercial ELISA kits (Thermo Fisher Scientific, Shanghai, China), according to the manufacturer’s standardized protocols.

### 2.6. T Cell Flow Cytometry Analyses

Single-cell suspensions from the spleen and the MLN were resuspended in PBS (Solarbio, Beijing, China). Viability was assessed using the Zombie NIR™ Fixable Viability Kit (BioLegend, San Diego, CA, USA) via 15 min incubation at 4 °C. Cells were subsequently blocked with blocking antibody 2.4G2 (anti-FcγRIII/I) (BioLegend, San Diego, CA, USA) for another 15 min at 4 °C. Samples were then washed once in PBS containing 2% FBS (Cellmax, Beijing, China). For the detection of Th cells, cell surface-expressed molecules (CD4 and CD25) were stained with FITC-labeled anti-mouse CD4 (BioLegend, San Diego, USA) and PE-labeled anti-mouse CD25 (BioLegend, San Diego, CA, USA) for 30 min at 4 °C. Following one wash, the nuclear staining was performed by using the staining buffer kits (eBioscience, Thermo Fisher Scientific, St. Louis, MO, USA). And nuclear antigens were stained using Alexa Fluor 647-labeled anti-mouse Foxp3 (BioLegend, San Diego, CA, USA), Brilliant Violet™ 421 (BV421)-labeled anti-mouse GATA3 (BioLegend, San Diego, CA, USA), PE-Cyanine7 (PE-Cy7)-labeled anti-mouse T-bet (BioLegend, San Diego, CA, USA), and PerCP-eFluor 710 (PerCP-eF710)-labeled anti-mouse RORγt (BioLegend, San Diego, CA, USA) for 40 min at 4 °C. Cells were then washed once with 1 × PBS containing 2% FBS and immediately analyzed by the CytoFlex S flow cytometer (Beckman Coulter, Brea, CA, USA).

### 2.7. Tissue Staining and Observation

The jejunum samples were collected for histology analysis. The tissue samples were fixed in 4% paraformaldehyde at room temperature for 24 h, dehydrated in 70% ethanol at 4 °C for 72 h, and subsequently embedded in paraffin. Jejunum sections were stained with hematoxylin and eosin (H&E), and images were acquired using an optical microscope (DM2500, Leica, Wetzlar, Germany).

### 2.8. RNA Extraction and Analysis

Following isolation, jejuna were immediately stabilized in RNAlater Stabilization Solution (Servicebio, Wuhan, China), then stored at −20 °C until RNA extraction using RNAiso Plus reagent (TAKARA Bio, Kusatsu, Japan). cDNA was synthesized using the Reverse Transcription kit (Vazyme, Nanjing, China). qPCR was performed with ChamQ Universal SYBR qPCR Master Mix (Vazyme, Nanjing, China) on a QuantStudio™ 3 system (Applied Biosystems, Thermo Fisher Scientific, Waltham, MA, USA). qPCR primer sequences for target genes (*IL33*, *Occludin1*, ZO1 tight junction protein (*TJP1*), *RORγt*, and *Foxp3*) are listed in Table S3. Amplification protocols were performed in 40 cycles, according to the kit instructions, with gene expression normalized to Gapdh using the comparative Ct method (2^−ΔΔCt^ method).

### 2.9. Statistical Analyses

All statistical analyses were performed with GraphPad Prism 8 software. Results are shown as means ± SEM. For the analysis among the four groups, one-way ANOVA followed by Tukey’s post hoc test was used. For analyzing data involving two independent parameters, two-way ANOVA followed by Tukey’s post hoc test was used. * *p* < 0.05, ** *p* < 0.01, *** *p* < 0.001, and **** *p* < 0.0001 represent significant differences.

## 3. Results

### 3.1. HFD-Induced Mild Obesity Slightly Alleviates BLG-Induced Allergic Response in Female BALB/c Mice

In general, male mice are widely used in HFD-induced obesity models, whereas female BALB/c mice are more suitable for food allergy models [24,25]. We observed that HFD-fed female mice exhibited body weight gains of approximately 19.81% and 10.92% before and after food allergy induction, respectively (Figure 1A,B). Correspondingly, the weight of belly fat, serum total cholesterol (T-CHO), and low-density lipoprotein cholesterol (LDL-C) concentrations were significantly increased by 216.42%, 32.45%, and 39.50% in HFD-fed mice, respectively (Figure 1D–F). Notably, no significant differences in body weight, the concentration of fasting blood glucose, or serum lipid profiles were detected between allergic and non-allergic groups (Figure 1B–F). Collectively, these findings confirmed the successful establishment of a mild obesity model. Following the 4-week BLG immunization protocol outlined in Figure 1A, BLG-sensitized mice fed with the COD exhibited pronounced allergic symptoms and significant body temperature reductions relative to non-allergic mice fed with the COD (Figure 1G,H). Moreover, elevated anti-BLG IgG1 and mMCP-1 levels were detected in both COD- and HFD-fed allergic mice (Figure 1I,J). There was a lower level of anti-BLG IgG1 in HFD-fed allergic mice than in the mice fed with the COD, though this difference did not reach statistical significance. In contrast, HFD-fed allergic and non-allergic groups showed comparable symptom severity. Intriguingly, HFD intervention attenuated hypothermia in allergic mice. Furthermore, short-term HFD exposure (9 weeks) without obesity induction failed to alter immunopathological parameters (Figure S2). These results collectively indicated that HFD-induced mild obesity elicits attenuated allergic responses in murine models.

### 3.2. HFD Maintains Th2/Th1 Balance and Activates Th17 Cells in the Spleen

**Figure 2:**
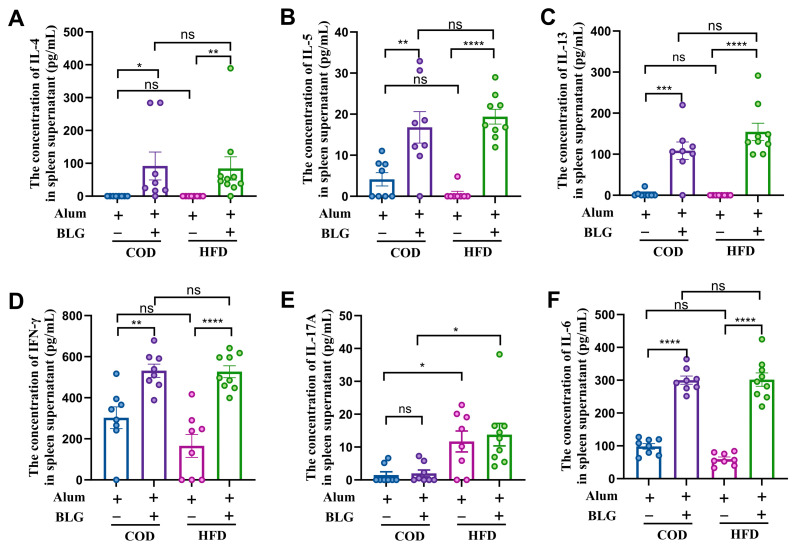
CD4^+^ T-associated cytokines ((**A**) IL-4; (**B**) IL-5; (**C**) IL-13; (**D**) IFN-γ; (**E**) IL-17A; (**F**) IL-6) production in the supernatants of BLG-restimulated spleen cells. HFD = high-fat diet; COD = control diet; BLG = β-lactoglobulin; ns = no significant. * *p* < 0.05, ** *p* < 0.01, *** *p* < 0.005, and **** *p* < 0.001.

The spleen is a critical peripheral lymphoid organ for antigen-specific T cell responses, essential for initiating systemic immunity and intestinal allergic responses [26]. To investigate the impact of HFD-induced mild obesity on systemic cellular immunity, we examined Th1-, Th2-, and Th17-related cytokine production in BLG-restimulated splenocytes ex vivo (Figure 2). Splenocytes from allergic mice produced elevated BLG-specific Th1 and Th2 cytokines upon restimulation (Figure 2A–D). The allergic mice fed with an HFD produced high levels of IL-4, IL-5, and IL-13 compared to allergic mice fed with the COD, though without statistical significance (Figure 2A–C). Notably, BLG-restimulated splenocytes from HFD-allergic mice exhibited significantly increased IL-17A production (Figure 2E). As Th17 differentiation requires IL-6-STAT3 signaling [27], we observed significantly elevated IL-6 in allergic mice, with no differences between the HFD-fed group and the COD-fed group (Figure 2F).

**Figure 3:**
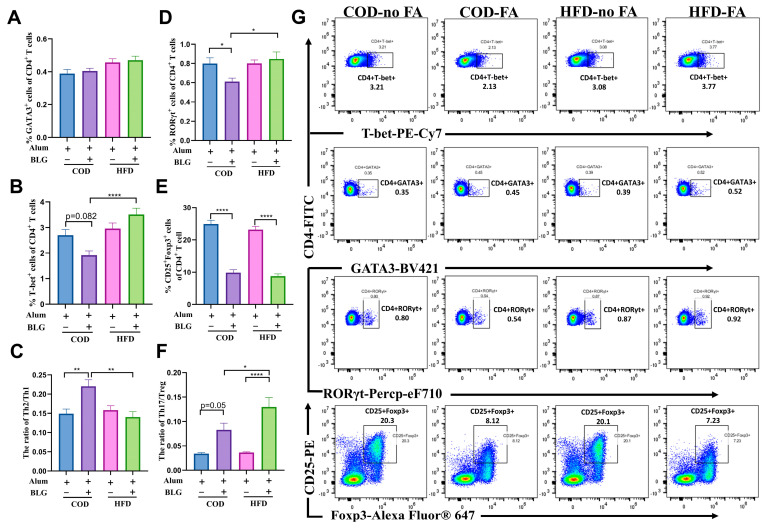
HFD maintains the Th2/Th1 balance and activates Th17 cells in the spleen. The quantification of Th2 cells (**A**), Th1 cells (**B**), Th17 cells (**D**), and Treg cells (**E**) in the spleen. (**C**) The ratio of Th2/Th1 in the spleen. (**F**) The ratio of Th17/Treg in the spleen. (**G**) For quantification, Th1 cells were gated as live, CD4, and T-bet; Th2 cells were gated as live, CD4, and GATA3; Th17 cells were gated as live, CD4, and RORγt; Treg cells were gated as live, CD4, CD25, and Foxp3. HFD = high-fat diet; COD = control diet; BLG = β-lactoglobulin; FA = food allergy; ns = no significant. * *p* < 0.05, ** *p* < 0.01, and **** *p* < 0.001.

In addition, we analyzed CD4^+^ T cell differentiation using flow cytometry (Figure 3). A slight increase in CD4^+^GATA3^+^ (Th2) cells was observed between allergic mice fed with the COD versus HFD, though without statistical significance (Figure 3A). The percentage of CD4^+^T-bet^+^ (Th1) cells significantly increased by 102.32% in HFD-fed allergic mice compared to COD-fed allergic mice (Figure 3B). The Th2/Th1 ratio was markedly reduced by 28.41% in HFD-fed allergic mice compared to allergic mice fed with the COD (Figure 3C), suggesting that the HFD alleviates BLG-induced Th2/Th1 imbalance through splenic Th1 cell expansion. Moreover, CD4^+^RORγt^+^ T (Th17) cells in the spleen of allergic mice fed with the HFD were significantly increased by 38.69% (Figure 3D). This result corresponds to the change in IL-17A above. Furthermore, the frequency of CD4^+^CD25^+^Foxp3^+^ T (Treg) cells was significantly decreased in allergic mice, and there was no significant difference between allergic mice fed with the HFD and COD (Figure 3E). The Th17/Treg cell ratio significantly increased by 56.67% in allergic mice fed with the HFD (Figure 3F). Gating strategies for the imaging flow cytometry analysis are found in Figure 3G.

### 3.3. HFD Does Not Alter CD4^+^ T Cell Activation in the MLN

**Figure 4:**
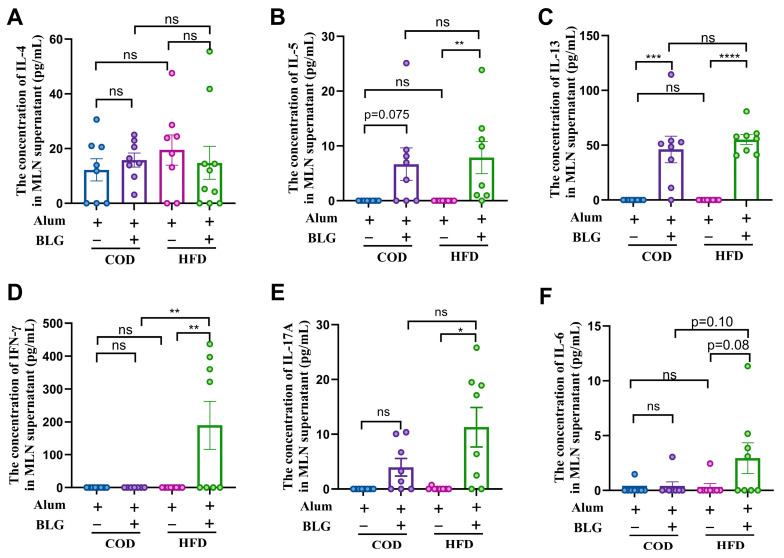
CD4^+^ T-associated cytokine ((**A**) IL-4; (**B**) IL-5; (**C**) IL-13; (**D**) IFN-γ; (**E**) IL-17A; (**F**) IL-6) production in the supernatants of BLG-restimulated the MLN cells. HFD = high-fat diet; COD = control diet; BLG = β-lactoglobulin; ns = no significant. * *p* < 0.05, ** *p* < 0.01, *** *p* < 0.005, and **** *p* < 0.001.

MLN serves as an important inductive site for CD4^+^ T cell activation in the pathogenesis of food allergies [22,28]. To study the impact of HFD-induced obesity on the cellular response in the MLN, we tested the production of Th1-, Th2-, and Th17-related cytokines upon BLG-restimulated MLN cells ex vivo (Figure 4). MLN cells from BLG-sensitized mice exhibited a significantly elevated production of Th2-related cytokines (IL-4, IL-5, and IL-13), whereas no significant differences in cytokine profiles were observed between the COD-fed and HFD-fed allergic mice groups (Figure 4A–C). Notably, HFD-fed allergic mice demonstrated significantly elevated levels of IFN-γ, IL-17A, and IL-6 in MLN supernatants following BLG restimulation (Figure 4D–F). Intriguingly, HFD feeding in the absence of obesity did not modulate BLG-driven Th2 polarization but selectively elevated IL-17A production in antigen-restimulated MLN cells (Figure S2).

**Figure 5:**
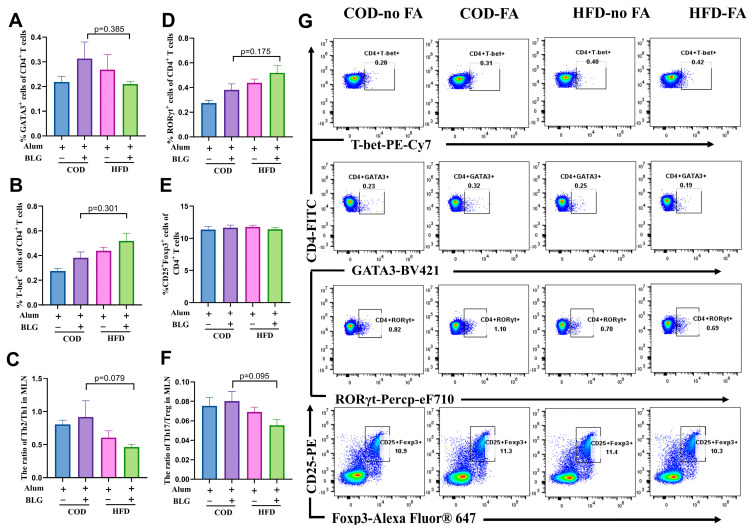
HFD does not alter CD4^+^ T cell activation in the MLN. Quantification of Th2 cells (**A**), Th1 cells (**B**), Th17 cells (**D**), and Treg cells (**E**) in the MLN. (**C**) The ratio of Th2/Th1 in the MLN. (**F**) The ratio of Th17/Treg in the MLN. (**G**) For quantification, Th1 cells were gated as live, CD4, and T-bet; Th2 cells were gated as live, CD4, and GATA3; Th17 cells were gated as live, CD4, and RORγt; Treg cells were gated as live CD4, CD25, and Foxp3. HFD = high-fat diet; COD = control diet; BLG = β-lactoglobulin; FA = food allergy.

A flow cytometry analysis revealed a lower percentage of CD4^+^GATA3^+^ T(Th2) cells and a higher percentage of CD4^+^T-bet^+^ T(Th1) cells in allergic mice fed with an HFD than in allergic mice fed with a COD (Figure 5A,B), which is also consistent with the data from the spleen. Moreover, we found that HFD-fed allergic mice exhibited not only a reduction in the Th2/Th1 ratio compared to COD-fed allergic mice (Figure 5C) but also a suppression of the Th17 responses and a decrease in the Th17/Treg ratio (Figure 5D–F). Gating strategies for the imaging flow cytometry analysis are found in Figure S1.

### 3.4. HFD Regulates Th17/Treg Balance in the Small Intestine and Contributes to Intestinal Homeostasis

**Figure 6:**
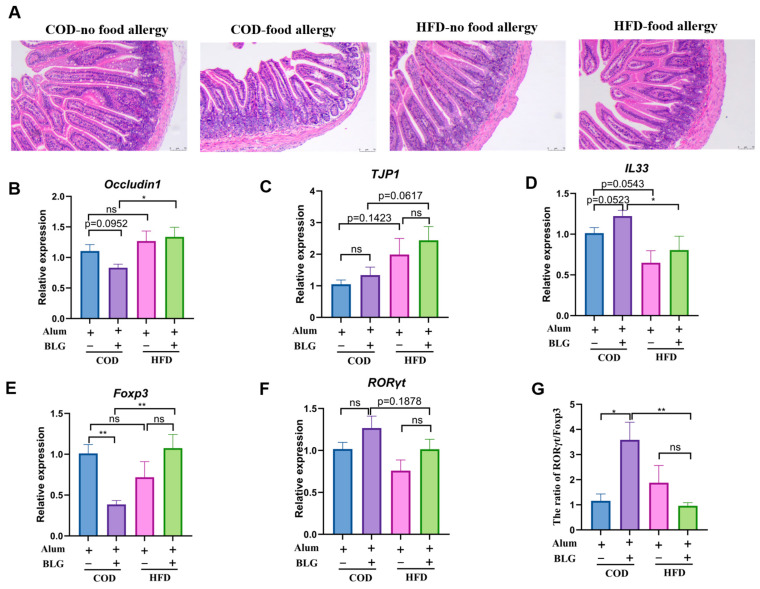
HFD maintains intestinal homeostasis. (**A**) Small-intestine (jejunum) histologic analysis; scale bar = 75 μm. The relative gene expression of *Occludin1* (**B**), *TJP1* (**C**), *IL33* (**D**), *Foxp3* (**E**), and *RORγt* (**F**), and the expression ratio of *RORγt* and *Foxp3* (**G**) determined in the jejunum of allergic mice fed with the HFD. HFD = high-fat diet; COD = control diet; BLG = β-lactoglobulin; ns = no significant. * *p* < 0.05 and ** *p* < 0.01.

The small intestine harbors diverse immune cell populations within the epithelial barrier and lamina propria that collectively defend against foreign allergens. Intestinal epithelial cells (IECs) form a physical–chemical barrier, which acts as the primary ‘gatekeeper’. Through the histological examination of intestinal architecture, we evaluated how the HFD modulates food allergy-induced morphological changes. Compared to the control group, allergic mice exhibited structurally disordered intestinal villi with evident atrophy (Figure 6A). However, the HFD did not significantly alter intestinal integrity in allergic mice. Notably, the HFD upregulated *Occludin1* and *TJP1* expression, with HFD-fed allergic mice showing significantly higher *TJP1* levels than COD-fed allergic mice (Figure 6B,C).

IL-33, secreted by intestinal epithelial cells, plays a critical role in orchestrating Th2-mediated immune responses [29]. As demonstrated in Figure 6D, IL-33 expression was marginally elevated in allergic groups compared to non-allergic controls. Interestingly, the expression of *IL33* was inhibited in both the HFD-fed allergic group and the non-allergic group. We further assessed the impact of the HFD on T cell differentiation within the jejunum. Allergic HFD-fed mice exhibited a reduced expression of *RORγt* (Th17 cell-specific transcription factor) and enhanced *Foxp3* (Treg cell-specific transcription factor) expression relative to COD-fed allergic mice (Figure 6E,F). In addition, notably, the HFD restored physiological *RORγt*/*Foxp3* ratios in allergic mouse intestines (Figure 6G). These results suggested that HFD-induced mild obesity restored the balance of Th17/Treg in the intestine, potentially preserving barrier integrity and mucosal homeostasis.

## 4. Discussion

Current epidemiological data provide a basis for investigating the relationship between high-fat dietary intake, obesity, and food allergies. In this study, we found that HFD-induced mild obesity promoted Th1-, Th2-, and Th17-mediated immune responses in a BLG-induced food allergy. HFD-induced obesity mildly affected food allergy symptoms, potentially through the regulation of the Th2/Th1 balance in the spleen and the Th17/Treg ratio in the intestine.

While epidemiological studies suggested that the HFD exacerbates type 2 immunity-mediated allergic disorders [30], our experimental models revealed that such outcomes may be modulated by sexual dimorphism and a degree of obesity. Specifically, male BALB/c mice developing severe obesity (body weight increase over 35%, accompanied by elevated fasting glucose) after 15 weeks of HFD exposure exhibited no changes in the activation of Th cells in the spleen, the suppression of Tregs in the MLN, and the activation of gut mucosal mast cells [31], consistent with prior evidence that hyperglycemia disrupts oral tolerance via impaired Treg differentiation [32]. In contrast, 18-week HFD-induced mild obesity in BALB/c female mice failed to amplify food allergy susceptibility, which might be partly due to the preservation of glycemic homeostasis.

In our study, HFD-fed allergic mice demonstrated significantly promoted splenic Th17 cell activation and alleviated food allergy symptoms. This aligns with reports of markedly increased CD4+IL-17A+ Th17 cells in obese individuals’ peripheral blood [33]. Moreover, IL-17A reduction and attenuated food allergen-responsive Th17 cells in IgE-mediated food allergy patients suggest a protective correlation between Th17 augmentation and allergy prevention [21]. Paradoxically, Th17 activation in the lung, skin, and gut has been shown to exacerbate food allergen-induced allergic inflammation [34,35]. Studies using OVA-sensitized models have demonstrated that Th17/Treg rebalancing ameliorates allergic inflammation [36]. Previous work revealed reduced Th17 but elevated Th1 proportions in intestinal tissues from obese mice [37]. Consistent with this study, we found that HFD-induced obesity biased immunity toward Th1 dominance and promoted Th17/Treg balance in the intestinal system, potentially contributing to alleviated BLG-induced allergic symptoms.

The occurrence and development of food allergies are closely related to the destruction of the intestinal barrier. The HFD reduced the production of IL-17A, which led to impaired intestinal barrier integrity [38]. However, a large amount of data confirmed that IL-17A contributes to promoting intestinal barrier repair [39,40]. In our study, we observed the elevated IL-17A production in food allergen-restimulated MLN cells from mildly allergic obese mice. HFD-induced mild obesity participates in regulating the Th17/Treg balance, a mechanism crucial to intestinal homeostasis [41]. HFD-induced mild obesity increased the transcription of *Occludin1* and *TJP1*, key markers of barrier integrity [42]. These findings suggest that HFD-induced IL-17A and Th17/Treg balance may protect the intestinal barrier in allergic mice.

Intestinal epithelial cells secrete IL-33 as an intestinal stress alarmin in response to barrier damage during allergen-induced allergic inflammation. High intestinal IL-33 expression facilitates and regulates innate immune responses, playing a crucial role in activating Th2-mediated immunity [43]. Consistent with this, allergic mice exhibited elevated intestinal IL-33 expression compared to non-allergic controls. However, our HFD-induced obesity model showed significantly decreased IL-33 expression. Previous clinical studies revealed an inverse correlation between IL-33 levels and body weight in lean/overweight populations [44]. Nevertheless, recent work found that the circulating level of IL-33 is upregulated in obese individuals [45]. The decrease in body weight after establishing the food allergy model might lead to the downregulation of IL-33 levels in mice fed with the HFD. Therefore, maintaining the state of obesity might be a key risk factor for allergic disease. Surprisingly, the IL-33/ST2 signaling pathway appears dispensable for BLG-induced Th2 responses, as IL-33 reduction in HFD-fed allergic mice did not alter type 2 immunity intensity. Emerging evidence implicates this axis in Th17-mediated immunity [46]. Corresponding to this, we also found the increased IL-33 in tandem with the increase in Th17 cells in the intestine. Therefore, IL-33/ST2 axis inhibition may restore Th17/Treg equilibrium to maintain intestinal homeostasis.

## 5. Conclusions

Our data demonstrated that HFD-induced mild obesity in female BALB/c mice significantly enhanced splenic Th1 and Th17 cell proportions, which contributed to the modest amelioration of IgE-mediated cow’s milk allergy symptoms. Concurrently, HFD-induced metabolic changes reduced intestinal Th17 cell frequency and the Th17/Treg ratio, potentially facilitating the maintenance of intestinal homeostasis. These immunopathological modifications ultimately prevented overt allergic symptom manifestation in sensitized mice. In summary, our results indicated that prolonged HFD exposure in female BALB/c mice did not exacerbate BLG-induced allergic reactions.

## 6. Limitations of This Study

The experimental design employing female BALB/c mice to establish an obesity model for investigating the obesity–food allergy association presents several inherent limitations. Firstly, strain-specific susceptibility to HFD-induced obesity varies substantially, with BALB/c mice exhibiting greater resistance to obesity-associated metabolic alterations compared to C57BL/6J counterparts [47]. Secondly, estrogen secretion in female mice substantially interferes with obesity induction [48], potentially explaining the modest obese phenotype observed despite prolonged 18-week HFD feeding. Given that males constitute a predominant proportion of global obesity cases and emerging evidence of sexual dimorphism in HFD-induced immunometabolism between metabolic dysregulation and food allergy susceptibility [49,50], the current single-sex/strain analytical framework may inadequately elucidate dietary impacts on allergic responses. Future investigations should employ sex/strain comparative models integrated with metabolomic technologies to delineate sex/strain-dependent pathogenic mechanisms underlying obesity-associated food allergies. Furthermore, existing studies have demonstrated that the HFD modulates the development of food allergies by altering the gut microbiota structure and composition [8]. Consequently, future investigations should employ germ-free murine models to further elucidate the impact of HFD-induced obesity on BLG-triggered allergic immune responses.

## Data Availability

The original contributions presented in the study are included in the article/Supplementary Materials, further inquiries can be directed to the corresponding authors.

## Supplementary Materials

The following supporting information can be downloaded at: https://www.mdpi.com/article/10.3390/foods14111852/s1, Table S1. Experimental diet composition; Table S2. Scores assigned to trigger the symptoms following the oral challenge; Table S3. Quantitative PCR Primer Sets; Figure S1. Gating strategy for flow cytometer analysis; Figure S2. Short-term (9 weeks) HFD feeding in mice dose not induce obesity and alter the immune effects in BLG-induced food allergy.
