# Supplementary material for: High-Fat Diet-Induced Mild Obesity Alters the Activation of T Cells and Maintains Intestinal Homeostasis in Food Allergy Animal Model
Source: Foods. 2025 May 23;14(11):1852. doi: 10.3390/foods14111852 (PMC12154341; doi:10.3390/foods14111852)
Supplement: Supplementary file 1 [file foods-14-01852-s001.zip › foods-3628988-supplementary.pdf]

Supplementary Materials

Table S1. Experimental diet composition.

Nutrition Ingredients	10% kcal% Fat (COD)		60% kcal% Fat (HFD)	
	(g)	(kcal)	(g)	(kcal)
Isolated soy protein	200	800	200	800
L-Cystine	3	12	3	12
Corn starch	506.2	2024.8	0	0
Maltodextrin	125	500	125	500
Sucrose	68.8	275	68.8	275
Cellulose, BM200	50	0	50	0
Soybean Oil	25	225	25	225
Lard	20	180	245	2205
Mineral Mix S10026	10	0	10	0
Dicalcium Phosphate	13	0	13	0
Calcium Carbonate	5.5	0	5.5	0
Potassium Citrate	16.5	0	16.5	0
Vitamin Mix V10001	10	40	10	40
Choline Bitartrate	2	0	2	0
Total	1055	4057	773.8	4057
kcal/g diet	3.84		5.24	

Table S2. Scores assigned to trigger the symptoms following the oral challenge.

Score	Description
0	No symptoms
1	Scratching and rubbing around the snout and head
2	Loose stool
3	Immobility after prodding, tremors, and/or significant respiratory distress
4	Diarrhea

Table S3. Quantitative PCR Primer Sets.

Gene name	Forward primer (5' to 3')	Reverse primer (5' to 3')
GADPH	CCTGTTGCTGTAGCCGTATTCA	CCAGGTTGTCTCCTGCGACTT
IL-33	GCTGCGTCTGTTGACACATTGAG	GGTCTTGCTCTTGGTCTTTTCCAG
TJP1	CGAGGCATCATCCCAAATAAGAAC	TCCAGAAGTCTGCCCGATCAC
RORγt	ACAAATTGAAGTGATCCCTTGC	GGAGTAGGCCACATTACACTG
Foxp3	CCACGGGCACTATCACACAT	TTGCTTGAGGCTGCGTATGA
Occludin1	TTGAAAGTCCACCTCCTTACAGA	CCGGATAAAAAGAGTACGCTGG

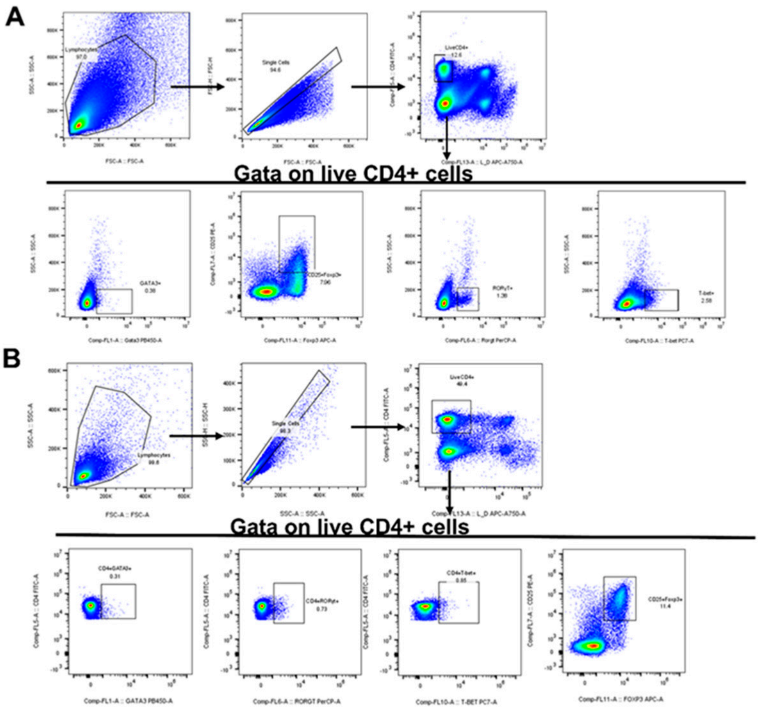

Figure S1. Gating strategy for flow cytometer analysis. (A) Gating strategy of T helper cells in the spleen. (B) Gating strategy of T helper cells in the MLNs.

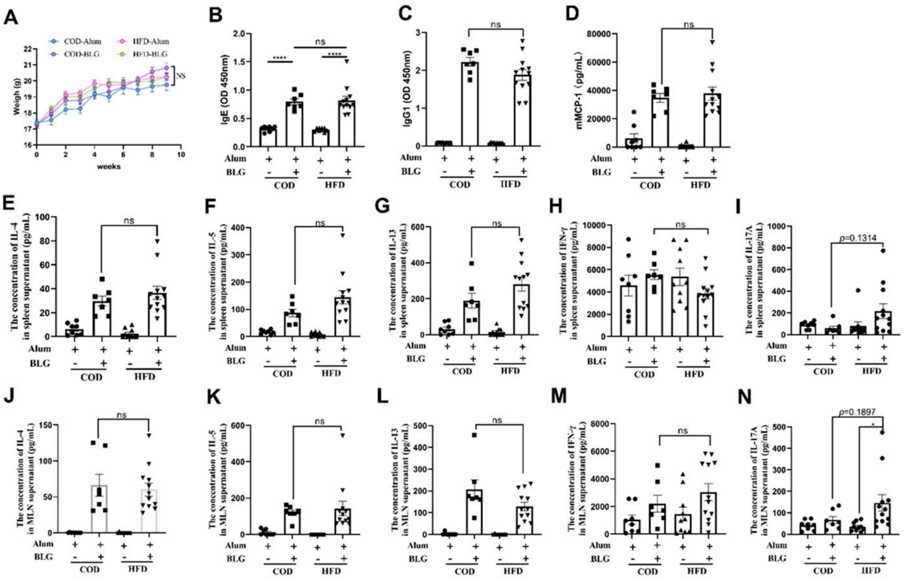

Figure S2. Short-term (9 weeks) HFD feeding in mice dose not induce obesity and alter the immune effects in BLG-induced food allergy. (A) The change of body weight. The BLG specific IgE (B) and IgG1 (C) levels. (D) The mMCP-1 level. The level of IL-4 (E), IL-5 (F), IL-13 (G), IFN-γ (H), IL-17A (I) from BLG-restimulated the spleen cells. The level of IL-4 (J), IL-5 (K), IL-13 (L), IFN-γ (M), IL-17A (N) from BLG-restimulated the MLN cells.
